# Impact of Potential Case Misclassification by Administrative Diagnostic Codes on Outcome Assessment of Observational Study for People Who Inject Drugs

**DOI:** 10.1093/ofid/ofae030

**Published:** 2024-01-16

**Authors:** David Goodman-Meza, Michihiko Goto, Anabel Salimian, Steven Shoptaw, Alex A T Bui, Adam J Gordon, Matthew B Goetz

**Affiliations:** Division of Infectious Diseases, David Geffen School of Medicine at UCLA, Los Angeles, California, USA; David Geffen School of Medicine at UCLA, Los Angeles, California, USA; Greater Los Angeles Veterans Health Administration, Los Angeles, California, USA; University of Iowa, Iowa City, Iowa, USA; Iowa City VA Medical Center, Iowa City, Iowa, USA; Division of Infectious Diseases, David Geffen School of Medicine at UCLA, Los Angeles, California, USA; Department of Family Medicine, David Geffen School of Medicine at UCLA, Los Angeles, California, USA; Medical & Imaging Informatics (MII) Group, Department of Radiological Sciences, UCLA, Los Angeles, California, USA; Informatics, Decision-Enhancement, and Analytic Sciences (IDEAS) Center, VA Salt Lake City Health Care System, Salt Lake City, Utah, USA; Program for Addiction Research, Clinical Care, Knowledge, and Advocacy (PARCKA), Division of Epidemiology, Department of Internal Medicine, University of Utah School of Medicine, Salt Lake City, Utah, USA; David Geffen School of Medicine at UCLA, Los Angeles, California, USA; Greater Los Angeles Veterans Health Administration, Los Angeles, California, USA

## Abstract

**Introduction:**

Initiation of medications for opioid use disorder (MOUD) within the hospital setting may improve outcomes for people who inject drugs (PWID) hospitalized because of an infection. Many studies used International Classification of Diseases (ICD) codes to identify PWID, although these may be misclassified and thus, inaccurate. We hypothesized that bias from misclassification of PWID using ICD codes may impact analyses of MOUD outcomes.

**Methods:**

We analyzed a cohort of 36 868 cases of patients diagnosed with *Staphylococcus aureus* bacteremia at 124 US Veterans Health Administration hospitals between 2003 and 2014. To identify PWID, we implemented an ICD code–based algorithm and a natural language processing (NLP) algorithm for classification of admission notes. We analyzed outcomes of prescribing MOUD as an inpatient using both approaches. Our primary outcome was 365-day all-cause mortality. We fit mixed-effects Cox regression models with receipt or not of MOUD during the index hospitalization as the primary predictor and 365-day mortality as the outcome.

**Results:**

NLP identified 2389 cases as PWID, whereas ICD codes identified 6804 cases as PWID. In the cohort identified by NLP, receipt of inpatient MOUD was associated with a protective effect on 365-day survival (adjusted hazard ratio, 0.48; 95% confidence interval, .29–.81; *P* < .01) compared with those not receiving MOUD. There was no significant effect of MOUD receipt in the cohort identified by ICD codes (adjusted hazard ratio, 1.00; 95% confidence interval, .77–1.30; *P* = .99).

**Conclusions:**

MOUD was protective of all-cause mortality when NLP was used to identify PWID, but not significant when ICD codes were used to identify the analytic subjects.

Medications for opioid use disorder (MOUDs), formulations of methadone, buprenorphine, and naltrexone are evidence-based interventions to improve health outcomes in people who inject drugs (PWIDs) and have opioid use disorder (OUD) [1]. PWIDs may inject multiple substances in isolation or in combination that includes opioids, methamphetamines, and cocaine, with opioids being the most frequently injected drug reported [2, 3]. PWIDs are at risk for bacterial infections that lead to hospitalizations such as skin and soft-tissue infections, endocarditis, and osteomyelitis. As such, there has been a call to initiate MOUD within hospital settings for PWIDs [4]. MOUD is important in treating PWIDs with OUD in hospital as those hospitalized for infectious diseases frequently leave when opioid withdrawal and cravings are not treated [5]. Yet, providers and hospitals may delay MOUD initiation until other medical issues are stabilized and the patient is seen in an outpatient setting either at opioid treatment programs or in settings that offer office-based addiction treatment with buprenorphine [6].

Research evaluating outcomes (eg, mortality, readmissions, patient-directed discharges) related to hospital-based initiation of MOUD in retrospective observational cohorts has been mixed [7–21]. Although multiple studies have reported improved health-related outcomes after starting MOUD within the hospital [11, 13, 21], multiple others have not. For example, 1 study using International Classification of Diseases, 10th edition (ICD-10), codes to define their analytic cohort showed that the initiation of MOUD in patients hospitalized with endocarditis had no effect on patient-directed discharges (also known as leaving against medical advice) and 30-day readmissions [14]. Ray et al found no significant difference in length of stay, readmissions, drug use at readmission, or reoperations between groups with and without MOUD [16]. O’Kane et al showed no statistical difference in all-cause 180-day readmission or mortality when prescribing buprenorphine at discharge [12]. Traver et al showed no association with the completion of outpatient parenteral antimicrobial therapy [20]. In this study, the authors acknowledged the possible selection bias in the use of ICD codes in potentially missing patients with incomplete documentation as well as the inclusion of patients who use prescription opioids and do not have a substance use disorder.

A major barrier to understanding the impact of MOUD on infectious diseases outcomes are imperfect systems of cohort identification [15, 22–26]. Many of these recent studies suggest that there is no viable combination of ICD code algorithms effective in identifying PWIDs. The lack of uniformity in ICD-based algorithms in identification of PWIDs may lead to misclassification of the population and inaccurate conclusions. Misclassification may be due to several factors: there is no specific ICD code for injection drug use; the subjects may not have injection drug use; may not have OUD; codes may be carried over from prior admission; and the disease and use of opioids may no longer be active [27]. For example, Marks et al showed that ICD codes misclassified 56.4% of PWIDs in a cohort of 114 patients with infective endocarditis. When analyzing the benefits of MOUD in relation to patient-directed discharges and all-cause mortality, the use of ICD codes suggested no benefit of MOUD. However, when using a manual chart review, there was a protective effect in both outcomes [28].

Recognizing the methodology gaps, our team published an algorithm based on natural language processing (NLP) and machine learning to identify PWIDs in electronic health records [26]. In that study, we showed that our NLP/machine learning algorithm, which was validated by manual chart review, had a sensitivity of 92.6% and a specificity of 95.4% in correctly identifying PWIDs, and the ICD-code algorithm with maximum accuracy had a sensitivity of 92.0% and a specificity of 52.4%. NLP, a discipline within computer science, addresses the comprehension, interpretation, and production of oral or written communication. Its applications are diverse across numerous research tasks. In our previous study, we trained an algorithm to categorize unstructured text data, such as admission notes, as pertaining to PWIDs or not related to PWIDs. Building off our initial analysis, our objective was to evaluate the differences in the clinical effectiveness of MOUD on outcomes in PWID admitted to Veterans Health Administration (VHA) hospitals because of *Staphylococcus aureus* bacteremia when identifying a cohort of PWIDs by ICD codes or by using natural language processing. We hypothesized that the way a cohort of PWID was identified was associated with MOUD effectiveness.

## METHODS

### Data

We used a cohort of 36 868 cases of patients diagnosed with *S. aureus* bacteremia during an inpatient visit at 124 VHA hospitals between 1 January 2003 and 31 December 2014. Data were extracted from the Corporate Data Warehouse through Veterans Affairs Informatics and Computing Infrastructure [29]. Data analysis was approved by the UCLA institutional review board, VHA Greater Los Angeles Healthcare System Research & Development Committee, University of Iowa institutional review board, and Iowa City VHA Research & Development Committee. Informed consent was waived for this study.

### PWID Identification

Based on our previous work [26], we used the ICD code–based and NLP-based algorithm with the maximum classification accuracies to subset the larger cohort into cases identified as PWIDs. Our ICD code–based algorithm included codes for hepatitis C and substance use disorders (Table 1) because these were shown to have the best diagnostic metrics in our prior analysis [26]. Our NLP algorithm used text preprocessing, regular expressions, NegEx to remove negation, term frequency-inverse document frequency for numeric representation, and a random forest model for classification of admission notes. The algorithm was designed to identify cases related to injection drug use regardless of substance of use.

**Table 1.** ICD Codes Used to Identify PWIDs

Concept	ICD-9 Diagnosis Codes
Opioid use disorder	30400 Opioid type dependence, unspecified 30401 Opioid type dependence, continuous 30402 Opioid type dependence, episodic 30403 Opioid type dependence, in remission
Cocaine use disorder	30420 Cocaine dependence, unspecified 30421 Cocaine dependence, continuous 30422 Cocaine dependence, episodic 30423 Cocaine dependence, in remission
Stimulant (methamphetamine) use disorder	30440 Amphetamine and other psychostimulant dependence, unspecified 30441 Amphetamine and other psychostimulant dependence, continuous 30442 Amphetamine and other psychostimulant dependence, episodic 30443 Amphetamine and other psychostimulant dependence, in remission
HCV	07041 Acute hepatitis C with hepatic coma 07051 Acute hepatitis C without mention of hepatic coma 07044 Chronic hepatitis C with hepatic coma 07054 Chronic hepatitis C without mention of hepatic coma 07070 Unspecified viral hepatitis C without hepatic coma 07071 Unspecified viral hepatitis C with hepatic coma V0262 carrier or suspected carrier of infectious disease, viral hepatitis C

Abbreviations: HCV, hepatitis C virus; ICD, International Classification of Diseases; PWID, people who inject drugs.

### Covariates and Primary Predictor

For each case in the cohorts, we extracted demographic variables (age, gender, race, ethnicity, homelessness), date and hospital of the index hospitalization, discharge status (patient-directed discharge or other), readmission, blood culture data (methicillin-resistant *S. aureus* [MRSA] or non-MRSA), ICD-9 codes, and medication administration data. Race and ethnicity were categorized based on US census categories. Race was categorized as American Indian or Alaska Native, Asian, Black or African American, Native Hawaiian or Other Pacific Islander, White, or unknown. Ethnicity was categorized as Hispanic or Latino, not Hispanic or Latino, or unknown. During the cohort period, the VHA used ICD-9 codes (the VHA transitioned to ICD-10 on 1 October 2015) [30]. ICD-9 codes were used to identify chronic comorbidities based on algorithms from the Centers for Medicare & Medicaid Services’ Chronic Conditions Data Warehouse [31]. ICD-9 codes were used to identify concomitant infectious diseases (human immunodeficiency virus [HIV], hepatitis B and C), psychiatric disorders (anxiety, bipolar disorder, depression, posttraumatic stress disorder, schizophrenia), medical comorbidities (diabetes, chronic kidney disease, chronic liver diseases, cardiovascular disease, ischemic heart disease, heart failure, obesity). ICD-9 codes were used to calculate a weighted Elixhauser score [32]. Additionally, we included a series of care quality process measures as variables (appropriate antibiotic treatment, infectious disease consultation, and echocardiography). These process variables were defined in a previous publication [29].

We defined the primary predictor as the prescription or not of inpatient MOUD. Inpatient MOUD was defined as the prescription of methadone or buprenorphine at any time during the index hospitalization for *S. aureus* bacteremia (this includes during an emergency department visit and inpatient stay). We did not include naltrexone in the analysis because VHA guidelines preferred methadone and buprenorphine as first-line agents [33]. We reviewed all cases that were identified as PWIDs that received MOUD by either algorithm. We reviewed notes and dosing for MOUD and classified if MOUD was prescribed for OUD, chronic pain, or palliative care.

### Outcomes

The primary outcome was 365-day all-cause mortality from the date of discharge. Date of death was ascertained using the VHA Vital Status file that integrates VHA and non-VHA sources. Survival days were calculated from the discharge day for the index hospitalization for *S. aureus* bacteremia until the day of death or censored if death did not occur within the mortality time frame. Secondary outcomes were 30-day mortality, 365-day acute hospital readmission status, time to first readmission, and discharge status (patient-directed discharge or other).

### Statistical Analyses

All analyses were conducted in R 4.1.2 within the VINCI platform. We calculated descriptive statistics and compared covariates by those who were prescribed inpatient MOUD with those who did not by using χ^2^ tests or Wilcoxon rank-sum tests. We then performed 2 separate analyses: an epidemiologic analysis and a comparative effectiveness analysis.

In the epidemiologic analysis, we estimated the number of *S. aureus* bacteremia cases among PWIDs in the original Goto cohort using ICD codes and NLP. We calculated and plotted yearly rates per 100 000 veterans enrolled in the VHA [34].

In the comparative effectiveness analysis, we fit a mixed-effects Cox regression models for both the cohort identified using NLP and the other identified by ICD codes. The main predictor was receipt of MOUD (methadone or buprenorphine) during the index hospitalization for *S. aureus* bacteremia. The primary outcome was mortality at 365 days from the discharge date of the index hospitalization. Models were controlled for demographic variables, MRSA status, concomitant infectious diseases, psychiatric disorders, medical comorbidities, and process variables. Hospital of admission and year of admission were treated as random effects. We only included cases that were discharged alive from the acute care admission. This was done to address immortal time bias by excluding cases that died during their index admission and mitigate survival differences in time from admission to start of MOUD. Time 0 for both groups (received inpatient MOUD and not received inpatient MOUD) was the day of their discharge. Additionally, we performed bivariate analyses for MOUD on secondary outcomes that included mortality at 30 days, readmission at 365 days, time to first readmission, and patient self-directed discharges during the index hospitalization. Schoenfeld residuals testing was done to test the proportional hazard assumption for the models.

## RESULTS

**Figure 1.**
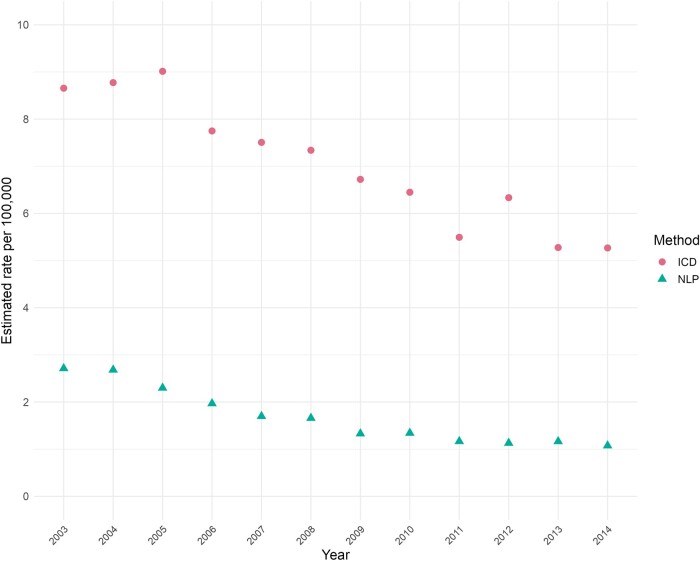
Estimated rates of *Staphylococcus aureus* bacteremia in people who inject drugs per 100 000 enrolled veterans in 124 hospitals in the Veterans Health Administration nationally from 2003 to 2014.

Of the 36 868 cases with *S. aureus* bacteremia, NLP identified 2389 (6.5%) cases as PWID, and ICD codes identified 6804 (18%) cases. Figure 1 displays epidemiologic trends of *S. aureus* bacteremia at the national level. The mean national rate per year over the study period of *S. aureus* bacteremia among PWIDs identified was 1.68 per 100 000 using NLP and 7.04 per 100 000 using ICD codes. Peak rates of *S. aureus* bacteremia were in 2004 and 2005 for the NLP and ICD methods, respectively. Rates decreased by 60% in the NLP cohort and by 41% in the ICD cohort at the end of the study period (2014).

For the comparative effectiveness analysis, we excluded 242 cases from the NLP cohort (10.1%) and excluded 730 cases from the ICD cohort (10.7%) because these cases died during the index admission. Our final analysis cohorts included 2147 cases in the NLP cohort and 6074 cases in the ICD cohort. There were 1636 cases that were present in both cohorts. MOUD were dispensed to 113 cases (106 methadone [93.8%], 7 buprenorphine [6.2%]) in the NLP cohort and 244 cases (234 methadone [95.6%], 10 buprenorphine [4.1%]) in the ICD code cohort (105 cases that received MOUD appeared in both cohorts). Of the 113 cases identified by NLP, 110 were confirmed to be PWIDs and have OUD (97.3%), 3 were prescribed for chronic pain and were not identified to be PWIDs (2.7%). Of the 244 cases identified by ICD codes, 122 were identified to be PWIDs and have OUD (50%), 95 had chronic pain (38.9%), 14 had palliative care indications (5.7%), and 13 others were identified as having OUD but not identified to be PWID (5.3%).

Table 2 shows demographic and clinical differences by MOUD status for both cohorts. In the NLP cohort, MOUD was less common in cases that identified as Black, had chronic kidney disease, and more common in those identified as Latino, diagnosed with endocarditis, hepatitis B and C, anxiety, and had chronic liver disease compared with those without a respective diagnosis. In the ICD cohort, MOUD was more common in cases identified as Latino, diagnosed with HIV, hepatitis C, endocarditis, osteomyelitis, and posttraumatic stress disorder, and less common in cases with schizophrenia, diabetes, or heart failure than those without a respective diagnosis.

**Table 2.** Descriptive Characteristics of People who Inject Drugs in a Cohort of Veterans Admitted for *Staphylococcus aureus* Bacteremia Between 2003 and 2014 Identified by Natural Language Processing or ICD Codes

Characteristic	NLP Cohort				ICD Cohort			
	Overall, N = 2147	MOUD, N = 113	No MOUD, N = 2034	*P* ^a^	Overall, N = 6074	MOUD, N = 244	No MOUD, N = 5830	*P* ^a^
Age at index admission, y	56 (51, 61)	55 (52, 59)	56 (51, 61)	.20	57 (53, 62)	56 (52, 59)	57 (53, 62)	<.001
Gender	…	…	…	.80	…	…	…	.60
Female	62 (2.9%)	2 (1.8%)	60 (2.9%)		143 (2.4%)	7 (2.9%)	136 (2.3%)	
Male	2085 (97%)	111 (98%)	1974 (97%)		5931 (98%)	237 (97%)	5694 (98%)	
Race	…	…	…	<.001	…	…	…	.30
White	1172 (55%)	66 (58%)	1106 (54%)		3463 (57%)	145 (59%)	3318 (57%)	
AIAN	24 (1.1%)	3 (2.7%)	21 (1.0%)		68 (1.1%)	1 (0.4%)	67 (1.1%)	
Asian	8 (0.4%)	2 (1.8%)	6 (0.3%)		8 (.1%)	1 (0.4%)	7 (0.1%)	
Black	754 (35%)	25 (22%)	729 (36%)		2066 (34%)	74 (30%)	1992 (34%)	
NHPI	27 (1.3%)	6 (5.3%)	21 (1.0%)		48 (0.8%)	3 (1.2%)	45 (0.8%)	
Unknown	162 (7.5%)	11 (9.7%)	151 (7.4%)		421 (6.9%)	20 (8.2%)	401 (6.9%)	
Ethnicity	…	…	…	.02	…	…	…	<.001
Latino	255 (12%)	23 (20%)	232 (11%)		530 (8.7%)	40 (16%)	490 (8.4%)	
Not Latino	1750 (82%)	82 (73%)	1668 (82%)		5113 (84%)	190 (78%)	4923 (84%)	
Unknown	142 (6.6%)	8 (7.1%)	134 (6.6%)		431 (7.1%)	14 (5.7%)	417 (7.2%)	
Homeless	604 (28%)	34 (30%)	570 (28%)	.60	1396 (23%)	67 (27%)	1329 (23%)	.09
MRSA	1023 (48%)	61 (54%)	962 (47%)	.20	3057 (50%)	126 (52%)	2931 (50%)	.70
HIV	892 (42%)	49 (43%)	843 (41%)	.70	1926 (32%)	110 (45%)	1816 (31%)	<.001
HBV	411 (19%)	34 (30%)	377 (19%)	<.01	1223 (20%)	59 (24%)	1164 (20%)	.11
HCV	1558 (73%)	103 (91%)	1455 (72%)	<.001	5039 (83%)	221 (91%)	4818 (83%)	.001
Associated infectious diagnosis								
Endocarditis	508 (24%)	41 (36%)	467 (23%)	.001	937 (15%)	62 (25%)	875 (15%)	<.001
Osteomyelitis	629 (29%)	38 (34%)	591 (29%)	.30	1723 (28%)	85 (35%)	1638 (28%)	.02
Septic Arthritis	267 (12%)	19 (17%)	248 (12%)	.15	648 (11%)	33 (14%)	615 (11%)	.14
Psychiatric diagnosis								
Anxiety	1132 (53%)	81 (72%)	1051 (52%)	<.001	3589 (59%)	157 (64%)	3432 (59%)	.09
Bipolar disorder	310 (14%)	17 (15%)	293 (14%)	.90	916 (15%)	39 (16%)	877 (15%)	.70
Depression	1122 (52%)	67 (59%)	1055 (52%)	.12	3480 (57%)	154 (63%)	3326 (57%)	.06
PTSD	474 (22%)	32 (28%)	442 (22%)	.10	1423 (23%)	70 (29%)	1353 (23%)	.05
Schizophrenia	144 (6.7%)	4 (3.5%)	140 (6.9%)	.20	523 (8.6%)	9 (3.7%)	514 (8.8%)	<.01
Medical diagnosis								
Diabetes	894 (42%)	42 (37%)	852 (42%)	.30	3084 (51%)	98 (40%)	2986 (51%)	<.001
CKD	1656 (77%)	79 (70%)	1577 (78%)	.06	4943 (81%)	187 (77%)	4756 (82%)	.05
Liver disease	1160 (54%)	69 (61%)	1091 (54%)	.12	3905 (64%)	157 (64%)	3748 (64%)	>.90
CVD	520 (24%)	22 (19%)	498 (24%)	.20	1805 (30%)	72 (30%)	1733 (30%)	>.90
IHD	1526 (71%)	85 (75%)	1441 (71%)	.30	4426 (73%)	185 (76%)	4241 (73%)	.30
Heart failure	502 (23%)	23 (20%)	479 (24%)	.40	1749 (29%)	55 (23%)	1694 (29%)	.03
Obesity	350 (16%)	26 (23%)	324 (16%)	.05	1182 (19%)	52 (21%)	1130 (19%)	.50
Elixhauser score	13 (5, 22)	14 (4, 21)	13 (5, 22)	>.9	17 (9, 26)	14 (6, 22)	17 (9, 26)	<.001

Values in table are median (IQR) or n (%).

Abbreviations: AIAN, American Indian or Alaska Native; CKD, chronic kidney diseases; CVD, cerebrovascular disease; HBV, hepatitis B virus; HCV, hepatitis C virus; ICD, International Classification of Diseases; IHD, ischemic heart disease; MOUD, medications for opioid use disorder; MRSA, methicillin-resistant *Staphylococcus aureus*; NHPI, Native Hawaiian and Pacific Islander; NLP, natural language processing; PTSD, posttraumatic stress disorder.

^a^Wilcoxon rank-sum test; Fisher exact test; Pearson's χ^2^ test.

Bivariate associations of MOUD with outcomes are described in Table 3. In the NLP cohort, cases that received MOUD were less statistically less likely to die at 365 days but not at 30 days and had a longer time to acute care hospital readmission compared with cases that did not receive MOUD. In the ICD code cohort, there was no difference in mortality, readmissions, time to readmission, or patient-directed discharges between those receiving MOUD or not.

**Table 3.** Outcomes of People who Inject Drugs in a Cohort of Veterans Admitted for *Staphylococcus aureus* Bacteremia Between 2003 and 2014 Identified by Natural Language Processing or ICD Codes

…			NLP Cohort			ICD Cohort		
Characteristic	Overall, N = 2147	MOUD, N = 113	No MOUD, N = 2034	*P* ^a^	Overall, N = 6074	MOUD, N = 244	No MOUD, N = 5830	*P* ^a^
Mortality, 30 d	717 (33%)	38 (34%)	679 (33%)	>.9	1665 (27%)	79 (32%)	1586 (27%)	.07
Mortality, 365 d	556 (35%)	17 (21%)	539 (36%)	<.01	1823 (38%)	60 (34%)	1763 (38%)	.20
Readmission, 365 d	1448 (67%)	84 (74%)	1364 (67%)	.11	4327 (71%)	182 (75%)	4145 (71%)	.20
Readmission time to first (d), median (IQR)	20 (0, 75)	42 (0, 127)	19 (0, 73)	.03	23 (3, 74)	26 (1, 102)	23 (3, 74)	.40
Patient-directed discharge	91 (4.2%)	2 (1.8%)	89 (4.4%)	.2	177 (2.9%)	5 (2.0%)	172 (3.0%)	.40

Numbers in table are either n (%) or median (IQR).

Abbreviations: ICD, International Classification of Diseases; IQR, interquartile range; NLP, natural language processing.

^a^Pearson χ^2^ test; Wilcoxon rank-sum test.

**Figure 2.**
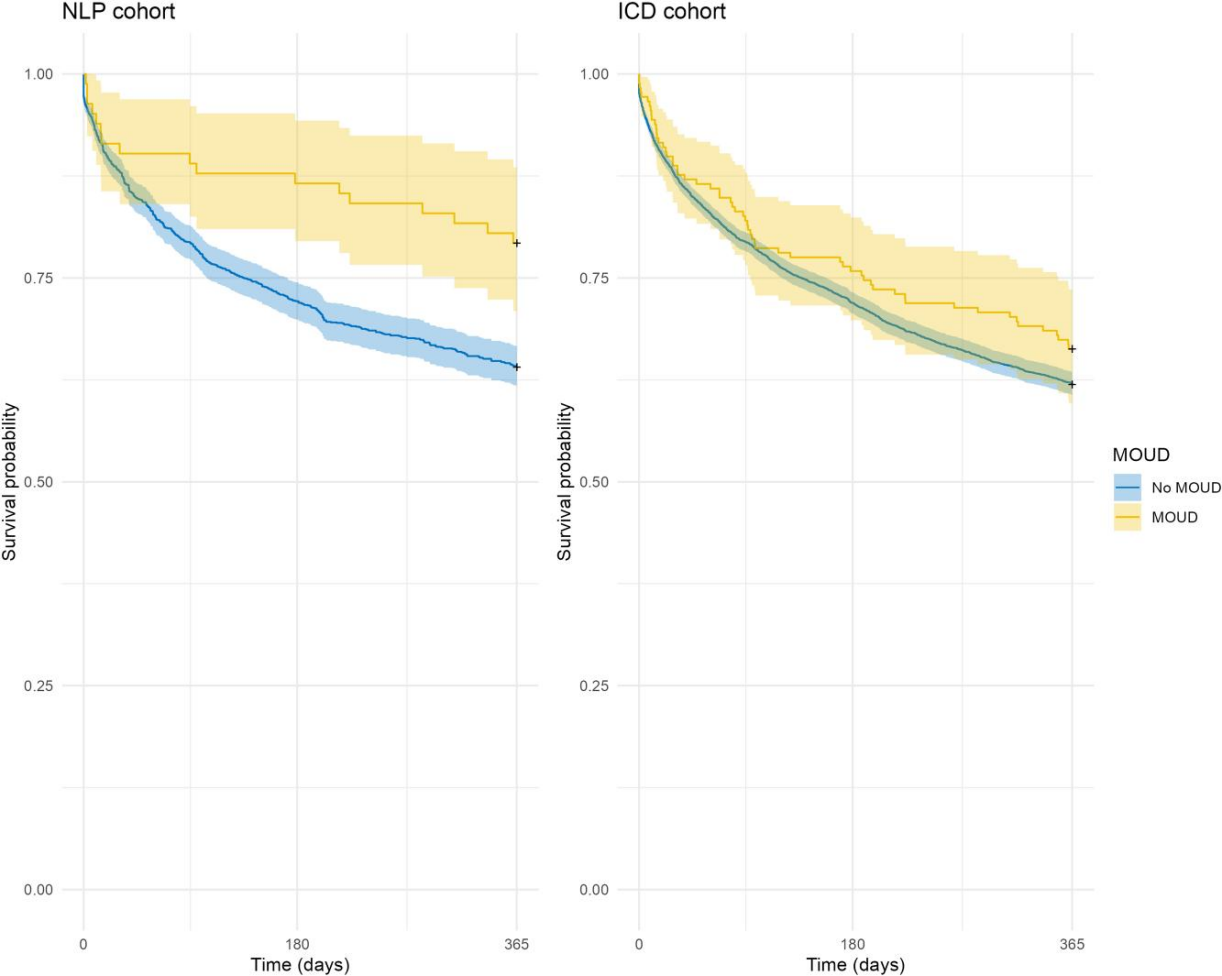
Survival probability over 1 y for people who inject drugs after hospitalization because of *Staphylococcus aureus* bacteremia by receipt of inpatient medications for opioid use disorders (MOUD).

In bivariate survival analysis, receipt of inpatient MOUD was associated with a protective effect on 365-day survival (hazard ratio [HR], 0.52; 95% confidence interval [CI], .32–.84; *P* < .01) compared with those not receiving MOUD in the NLP cohort. There was no significant effect of receipt of MOUD in the ICD cohort (HR, 0.85; 95% CI, .66–1.10; *P* = .02), as depicted in Figure 2. In multivariate survival analysis, receipt of MOUD retained its protective effect (adjusted HR [AHR], 0.48; 95% CI, .29–.81; *P* < .01) compared with not receiving MOUD after adjustment for covariates in the NLP cohort. Again, receipt of MOUD was not significant in the ICD cohort (AHR, 1.00; 95% CI, .77–1.30; *P* = .99). Schoenfeld tests were nonsignificant in both the NLP and ICD cohort analysis, suggesting that the proportional hazards assumption was not violated.

## DISCUSSION

We showed that the methods used to delineate a cohort of PWIDs in electronic health records had a significant impact on both epidemiologic and comparative effectiveness analyses. In the first, the ICD code–based method overestimated the rate of *S. aureus* bacteremia cases by more than 4-fold compared with NLP. In the second, the effectiveness of prescribing MOUD—more specifically, methadone—to PWIDs in the hospital was only shown to be beneficial when delineating a precise cohort using NLP, whereas when using an ICD code–based approach, the effect of MOUD was nonsignificant. The bias toward the null is most likely from misclassification. The ICD-based approach likely captured many cases that did not merit MOUD because of its low specificity and many who were receiving methadone for other reasons (eg, palliative care or chronic pain). To this point, 50% of the cases captured by ICD codes and received MOUD were not PWIDs.

Receipt of MOUD in the hospital was very low for PWIDs within the cohort. This is similar to what has been reported nationally for people living with OUD, where less than 20% receive any treatment for OUD and even less receive medication-based treatment [35]. MOUD prescriptions for buprenorphine or methadone historically are provided to more than 15% of veterans with OUD, but as of 2020 more than 40% of veterans receive formulations of MOUD (buprenorphine, methadone, and naltrexone) for OUD treatment at any given point [36]. Within the VHA in 2017, only 15% of admissions with OUD received MOUD and mostly for withdrawal management [37]. Only 2% received linkage to outpatient addiction care. Multiple efforts have been instituted nationwide to improve access to MOUD, and specifically within the VHA [38–40]. One specific effort in the VHA was the Stepped Care for Opioid Use Disorder, Train the Trainer [39]. Early outcomes form this program showed an increase in providers able to prescribe buprenorphine and an increase in MOUD in eligible patients by 4% [38, 40].

Our study underscores the importance of refining methods to identify PWIDs within healthcare records and support future efforts to use NLP to identify PWIDs. Nevertheless, NLP has limitations to widespread use, which include concerns about confidentiality when obtaining clinical notes and the need for extensive technical expertise and time are required to train, test, and evaluate models. Next steps include building on this work by enhancing NLP algorithms or further refining code-based approaches to accurately categorize PWID cohorts and developing real-time NLP algorithms to increase quality of care in hospitals for PWIDs. Newer large language models should be evaluated for the purpose of identifying PWIDs and for extracting richer data such as the type of substance used, the duration of use, the routes of usage, and treatment offered.

There are many limitations to this study. First, we only used receipt or not of MOUD during inpatient admissions. Many other factors related to MOUD may mediate the relationship with our analyzed outcomes. These may include the specific medication, dose, duration of treatment, and linkage to outpatient care. The majority of cases that received MOUD were prescribed methadone, and we cannot extrapolate our findings to the small number that received buprenorphine. Second, because PWIDs are a heterogenous group, cases that may have been exclusively using methamphetamines or cocaine may have been included in both groups; this may have biased the association of MOUD with the outcome toward the null. Further models should be developed to identify specific substances that PWIDs are reported to be using. Third, the study population was exclusively from the VHA and the findings may not generalize to other healthcare systems. As well, most cases were men, which may limit extrapolations of the results to other genders. Additionally, coding practices at the VHA may be different to other health systems. Fourth, our analyzed cohort was from before 2015, and this presents multiple limitations regarding comparability and applicability to contemporary cohorts. ICD-9 codes were used because those were current of the study period. Future studies need to compare NLP with ICD-10–based coding (and ICD-11 when it becomes available). During our study period, illicit fentanyl use was not commonly reported by PWIDs. Additionally, since then, there has been much more attention to improving inpatient diagnosis of substance use disorders and initiation of MOUD for people with OUD. These changes in substance use patterns and provision of care may change the relationship of MOUD with the outcome and the diagnostic metrics of both the ICD and NLP algorithms. Further evaluations of NLP to ICD codes with contemporary cohorts are necessary.

Identification of PWID in health services research is paramount to estimate the true benefits of different interventions in this population. The mortality rate of 29% at 1 year highlights the need for better interventions in PWIDs that are hospitalized for an infection, in this case *S. aureus* bacteremia. Continued efforts are needed to maximize the provision of evidence-based care measures for patients with *S. aureus* bacteremia such as appropriate antibiotics, infectious diseases consultation, echocardiography, and for PWIDs specifically, MOUD. Future research related to PWIDs should consider using NLP over ICD methods to identify cases to reduce bias resulting from misclassification.

## Notes

***Author contributions***. D. G. M., S. S., A. A. T. B., and M. B. G. conceptualized and designed the study. D. G. M. conducted the statistical analysis. M. G. assisted in obtaining access to the cohort data. All authors had full access to all data in the study and take responsibility for the integrity of the data and the accuracy of the data analysis. All authors contributed to the writing and critical revision of the report.

***Financial support*.** D. G. M. was supported by the US National Institute on Drug Abuse (grant K08 DA048163). S. S. was supported by the US National Institute on Mental Health (grant P30 MH58107), the UCLA Center for AIDS Research (grant 5P30 AI028697), and the UCLA Clinical Translational Science Institute (grant UL1 TR001881). All other authors have no conflicts of interest to declare and received no special funding related to this work.

***Data availability.*** Because of data use agreement limitations, source data are not exportable outside of the VHA. All analysis code will be available at https://github.com/davigood1/PWID_NLP on publication.
